# Misinterpretation of solid sphere equivalent refractive index measurements and smallest detectable diameters of extracellular vesicles by flow cytometry

**DOI:** 10.1038/s41598-021-03015-2

**Published:** 2021-12-17

**Authors:** Edwin van der Pol, Ton G. van Leeuwen, Xiaomei Yan

**Affiliations:** 1 Biomedical Engineering and Physics, Amsterdam University Medical Centers, location AMC, University of Amsterdam, Amsterdam, The Netherlands; 2 Laboratory Experimental Clinical Chemistry, Amsterdam University Medical Centers, location AMC, University of Amsterdam, Amsterdam, The Netherlands; 3 Vesicle Observation Center, Amsterdam University Medical Centers, location AMC, University of Amsterdam, Amsterdam, The Netherlands; 4 The MOE Key Laboratory of Spectrochemical Analysis & Instrumentation, The Key Laboratory for Chemical Biology of Fujian Province, Collaborative Innovation Center of Chemistry for Energy Materials, Department of Chemical Biology, College of Chemistry and Chemical Engineering, Xiamen University, Xiamen, 361005 Fujian People’s Republic of China

**Keywords:** Optical techniques, Biomarkers

**arising from**: G. C. Brittain IV et al.; *Scientific Reports* 10.1038/s41598-019-52366-4 (2019).

## Introduction

Flow cytometers are utilized to characterize single submicrometer particles in biofluids, such as extracellular vesicles (EVs) and viruses. However, because calibration of the optical signals measured by flow cytometers requires optical modelling and valid assumptions, which are not straightforward, statements in the literature about the sensitivity of flow cytometers are often either lacking or incorrect. In this Letter, we explain why measurement artifacts and a statistical artefact cause an overestimation of the refractive index of EVs as derived by Brittain et al.^1^. Because these artifacts lead to conclusions that are not supported by the data, we re-analyzed the data of Brittain et al. to rectify statements about the sensitivity of their flow cytometer.

The CytoFLEX used by Brittain et al. is a flow cytometer of which the optics have been designed before 2012^2^. The first peer-reviewed scientific publications using the CytoFLEX date from 2016^3,4^. The CytoFLEX houses several optical solutions, such as the catadioptric flow-cell design to maximize light collection, silicon avalanche photodiodes with higher light-detection sensitivity across a larger wavelength range than commonly used photomultiplier tubes, and wavelength-division multiplexing to limit signal loss, which were innovative at the time of introduction. Without any doubt, the optical solutions contribute to achieving single nanoparticle sensitivity, where every photon counts. However, the improvement attributed to the catadioptric flow-cell, as claimed by Brittain et al.^1^, must be nuanced. Whereas the full-width solid collection angle of the CytoFLEX is 110°, the full-width solid collection angle of other widely used flow cytometers, such as FACSCalibur, FACSCanto, FACSVerse, Gallios, Fortessa, and Navios, is ≥ 104°, but not 30°–60°, as the authors write.

Brittain et al. demonstrate detection of 70 nm polystyrene beads, 99 nm silica beads, and the 95 nm HAdV-5 virus using the violet side scatter detector. The sensitivity of the CytoFLEX is thereby similar to the flow cytometers developed by Hercher et al. in 1979^5^ and Steen in 2004^6^, who measured T2 bacteriophages and 74 nm polystyrene beads by scatter detection, respectively, but (99/24)﻿^6^ ≈ 4.9 × 10^3^ times less sensitive than the flow cytometer developed by Zhu et al.^7^, who measured 24 nm silica beads.

## Flow cytometry calibration to determine the refractive index of viruses

In the research field of extracellular vesicles (EVs), flow cytometry data are barely calibrated, leading to poor reproducibility of experiments and even detection of residual platelets instead of the anticipated EVs^8,9^. By calibrating the light scattering intensity, Brittain et al. improve data interpretation, confirm their results, and in addition enable data comparison, derivation of the refractive index (RI) from particles of known size, and derivation of the size from particles of known RI^10,11^. Nevertheless, the implementation of Mie theory by Brittain et al. is unconventional. Whereas Mie theory provides an analytical solution of Maxwell’s equations for spheres of all size parameters, Brittain et al. introduce an empirical function to take into account the angular scatter distribution, or phase function. The accuracy of the empirical method relies on the number of used reference beads. In addition, the empirical method lacks the opportunity to model core–shell particles, such as EVs. To overcome these limitations, we have solved Maxwell’s equations analytically and specifically for the CytoFLEX of Brittain et al., taking into account the optical configuration of their flow cytometer^10^.

**Figure 1:**
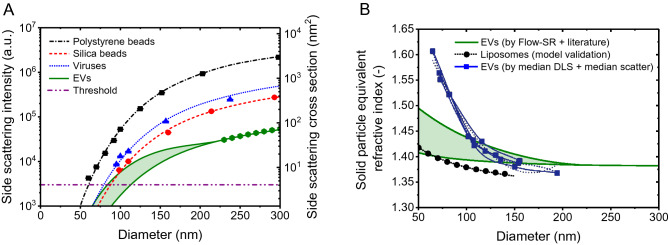
(**A**) Side scattering intensity measured (symbols) by a CytoFLEX^1^ and calculated (lines) by Mie theory^10^ versus diameter for polystyrene beads (squares), silica beads (circles), viruses (triangles), and CD61 + EVs (hexagons). The assumed refractive index (RI) is 1.627 for polystyrene beads, 1.440 for silica beads, and 1.472 for viruses at the illumination wavelength of 405 nm. EVs were modelled as core–shell particles, having a 12 nm thick shell with an RI of 1.52 and a core RI of 1.353 (upper solid line), and having a 4 nm thick shell with an RI of 1.450 and a core RI of 1.380 (lower solid line). The horizontal line indicates the trigger threshold. (**B**) Solid particle equivalent RI versus diameter for plasma-derived EVs based on Flow-SR^11^ and literature (panel A, solid green lines), for liposomes^19^ (dotted line), and for plasma-derived EVs based on the dynamic light scattering (DLS) measurements and the flow cytometry light scattering measurements performed by Brittain et al.^1^ (overlapping blue lines and symbols). Below a diameter of 100 nm, the solid particle equivalent RI based on DLS and flow cytometry light scattering is substantially higher compared to estimates based on Flow-SR^11^ and literature and liposome measurements.

Figure 1A shows the measured and calculated side scattering intensities versus diameter of the polystyrene beads, silica beads, and viruses measured by Brittain et al. The theory describes the measured scattering intensities of polystyrene beads perfectly (R^2^ = 0.9999), except for the 60 nm polystyrene beads. Whereas the model predicts a scattering intensity of 2974 arbitrary units for the 60 nm polystyrene beads, Brittain et al. measured 4225 arbitrary units, which is an overestimation of 42%. An overestimation of scatter signals close to the background noise is typical when using the height-parameter.

Based on the virus diameters and side scattering intensities from Brittain et al., we solved the RI of the viruses by least square fitting. We obtained an RI of 1.477 for HAdV-5, 1.488 for HIV-1, 1.471 for MLV, 1.468 for HSV-1, and 1.460 for Vaccinia at the illumination wavelength of 405 nm. The best fit through all virus datapoints resulted in an RI of 1.472, which is in concordance with Brittain et al. for two reasons. First, because viruses are monodisperse and studied for more than a century, virus diameters are well-known. Second, the light scattering intensities of the measured viruses are far above the trigger threshold (three to 82-fold) and detection limit. In our opinion, the shared RI of ~ 1.47 for different viruses is therefore the key finding of this manuscript. As confirmed by Brittain et al. (p. 6), the reversed approach, i.e. determining the size of the viruses from the estimated RI of ~ 1.47, result in the initial literature values. We agree with Brittain et al. that sizing particles by flow cytometry is an attractive application, because it is more precise than for example nanoparticle tracking analysis^12^ and allows better statistics than other detection techniques due to the inclusion of hundreds of thousands of particles.

## The spatial refractive index distribution of an extracellular vesicle

Next, Brittain et al. aim for estimating the RI of EVs. In contrast to beads, however, EVs do not have a homogenous RI distribution. Similar to cells^13^, the phospholipid membrane has a higher RI than the lumen of EVs. Therefore, EVs should be modelled as core–shell particles and the “RI of EVs” is rather the solid particle equivalent RI. We used the flow scatter ratio (Flow-SR)^11^ to measure the solid particle equivalent RI of *single* CD61 + EVs in plasma of 19 healthy individuals. For EVs with a diameter between 225 and 800 nm, which is the size range wherein Flow-SR is applicable for our flow-cytometer (A60-Micro, Apogee Flow Systems, UK), we found an RI of 1.383 ± 0.012 (median ± robust standard deviation), which is in agreement with other RI estimates of single EVs in plasma^14,15^ in this size range. The green hexagons in Fig. 1A show the anticipated side scattering intensities for solid particles with an RI of 1.383 for the CytoFLEX.

To validate our RI measurements and extrapolate our results to smaller diameters than 225 nm, we modelled EVs as core–shell particles, where we fixed the RI and thickness of the shell and solved the RI of the core to match the data. The lowest solid line in Fig. 1A represents the dimmest scenario, describing EVs having a 4 nm thick shell with an RI of 1.45. Please note that only phospholipid bilayers without proteins have a thickness of ~ 4 nm and that the lowest RI measured for phospholipid membranes is 1.45^16^. By least square fitting, we solved the RI of the core, which resulted in 1.380. An RI of 1.380 corresponds to a protein concentration (w/v) of ~ 25%^17^ and is a plausible RI for the lumen of cells and EVs^13^. Because the EVs were CD61 + and thus contained transmembrane proteins, the dimmest scenario probably underestimates the thickness and optical contribution of the membrane. The highest solid line in Fig. 1A therefore represents the brightest scenario, describing EVs having a 12 nm thick shell with an RI of 1.52. The RI of the shell is based on the highest value measured by fluorescence lifetime imaging microscopy in living cells^13^. By least square fitting, we solved the RI of the core, which resulted in 1.353. An RI of 1.353 corresponds to a protein concentration (w/v) of ~ 12%^17^ and is a plausible RI for the lumen of cells and EVs as well^18^. With molecular weights < 140 kDa, which corresponds to sphere equivalent diameters < 8 nm, antigens like CD61 and CD62p may increase the effective membrane thickness up to threefold. Most likely, however, the true scattering intensities of CD61 + EVs are in between the dimmest and the brightest scenario.

## Overestimation of the solid sphere equivalent refractive index of extracellular vesicles

In Fig. 1B, we related the scattering intensities of EVs, as calculated with the core–shell model, to their solid particle equivalent RI and diameter (green solid lines). Compared to Brittain et al., who suggest that the RI of 65 nm EVs could be up to 1.61 (overlapping blue lines), we found considerably lower values. To validate our approach, we compared the solid particle equivalent RIs for liposomes to earlier proposed literature values^19^ (black dotted line). In our opinion, the suggested RI of 1.61 for EVs is an overestimation, because it requires that EVs consist of pure proteins, which have RIs between 1.55 and 1.64^20^. However, proteins in or at EVs are hydrated and thus have a lower RI.

There are three explanations for the overestimation of the solid particle equivalent RI of CD61 + EVs by Brittain et al. First, a part of the measured scatter intensities of EVs is overestimated, because they are close to the detection limit and the height-parameter was used to analyze the data. This statement is confirmed by the 42% overestimation of the measured scattering intensity of 60 nm polystyrene beads in Fig. 1. Furthermore, the authors did not validate RI determination for particles with a scattering intensity close to the detection limit.

Second, determining the size of EVs in plasma samples by dynamic light scattering (DLS) should be avoided for two reasons. First, the samples are polydisperse (Fig. 6 in Ref.^1^) and “DLS is not suitable for PSD analysis of highly polydisperse samples”^21^. Although the EV samples had intermediate polydispersity (polydispersity index up to 0.5), the estimated median diameters require careful interpretation. Second, despite the application of size exclusion chromatography, the measured plasma samples contain a mixture of EVs and lipoproteins^11^. Because DLS is biased towards the presence of brightly scattering particles in a sample^22^, because lipoproteins have a higher RI than EVs^11^, and because lipoproteins likely outnumber EVs^23^, the median size determined by DLS was likely underestimated due to the presence of lipoproteins.

Third, the authors compare the median diameter of particles to the median side scattering intensity of CD61 + EVs, which causes an overestimation of the derived RI for two reasons. First, Fig. 6A^1^ and Supplementary Fig. S8^1^ show that the light scattering distributions of each EV population is truncated at the detection threshold, which causes an overestimation of the median side scattering intensity and hence an overestimation of the derived RI. Second, comparing the median diameter to the median side scattering intensity describe different particles, because the particle size distribution (PSD) and the scattering intensity distributions have different shapes and are skewed.

**Figure 2:**
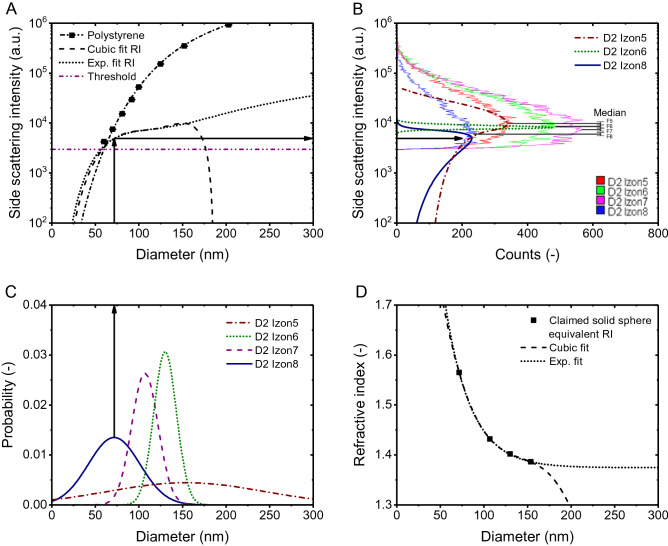
(**A**) Side scattering intensity measured (symbols) by a CytoFLEX^1^ and calculated (lines) by Mie theory^10^ versus diameter for polystyrene beads (squares). The assumed refractive index (RI) is 1.627 for polystyrene beads. EVs were modelled as particles with the diameter dependent solid particle equivalent RIs shown in panel D. The horizontal line indicates the trigger threshold. (**B**) Side scattering intensity of CD61 + EVs from fractionated plasma versus counts measured (light lines) by the CytoFLEX^1^ and predicted (dark lines) based on the measured size distribution in (**C**) and the scatter to diameter relation in (**A**) (black arrows). Colors indicate size exclusion chromatography fractions 5 (red and dash-dotted brown), 6 (green and dotted dark green), 7 (purple) and 8 (blue and solid dark blue). Fraction 7 resulted in a similar side scattering distribution as fraction 6 and is left out for clarity. Predictions do not describe the data. (C) Size distribution of particles from the same fractionated plasma as in panel B measured by dynamic light scattering (DLS; µ_Izon5_ = 153.82 nm, PDI_Izon5_ = 0.34, σ_Izon5_ = 89.7 nm, µ_Izon6_ = 130.17 nm, PDI_Izon6_ = 0.01, σ_Izon6_ = 13.0 nm, µ_Izon7_ = 106.99 nm, PDI_Izon7_ = 0.02, σ_Izon7_ = 15.1 nm, µ_Izon8_ = 71.66 nm, PDI_Izon8_ = 0.17, σ_Izon8_ = 29.5 nm). (**D**) Refractive index versus solid particle equivalent diameter claimed by Brittain et al. (symbols) fitted with a cubic function (y_0_ = 2.51109, A_1_ = − 0.02223, A_2_ = 1.51337E-4, A_3_ = 4, A_4_ = − 3.53334E-7) and with an exponential function (y_0_ = 1.37456, A = 2.12974, t_1_ = 29.67714).

Whereas we found a solid particle equivalent RI for EVs of 1.40–1.47 for 65 nm EVs and 1.39–1.40 for 150 nm EVs, Brittain et al. found 1.61 and 1.39, respectively. Because the three explained biases all add up to the uncertainty of the estimated RI, it is difficult to quantify the contribution of each bias to the overestimation of the RI. However, to reveal the main bias, the claimed relation between RI and diameter of EVs could be used to relate the measured size distributions to the measured scatter intensities. Figure 6A^1^ shows histograms of the measured scatter intensities of CD61 + EVs isolated by size exclusion chromatography (SEC) from plasma. Brittain et al. claim that the intensities indicated by the vertical lines labeled 8, 7, 6 and 5 represent EVs with increasing diameter (71.7 up to 153.8 nm) and decreasing RI (1.565 down to 1.386). If true, the PSDs in Fig. 6B^1^ could be related to the scatter intensities in Fig. 6A^1^ via the scatter to diameter relation, thereby incorporating the claimed RI to diameter relation in Fig. 6C^1^. To validate their approach, Fig. 2A of this Letter shows the scatter to diameter relation of CD61 + EVs as claimed by Brittain et al. We decided to fit the RI to diameter relation with the exponential decay function shown in Fig. 2D, because the cubic fit of the RI to diameter relation resulted in an asymptote.

We used the resulting scatter to diameter relation in Fig. 1A to relate the measured PSDs in Fig. 2C to the measured scatter intensities in Fig. 2B (black arrows). Figure 2B shows that the predicted scatter distributions do not describe the data. The overlap and width of the measured scatter intensities together with previously reported PSDs of CD61 + EVs^2,3^ and the working principle of SEC^1^ indicate that fractions 5 to 8 are polydisperse samples with overlapping PSDs. Because a scatter to diameter relation that would anticipate for the differences in polydispersity and PSDs measured by DLS does not exist, also not when taking into account the noise and precision of the flow cytometer, we believe that the use of DLS to analyze polydisperse samples is a main bias in the approach.

## Outlook and summary

To determine the true RI distribution of EVs < 100 nm, a sensitive, calibrated instrument, a validated procedure, and confirmation of results with an analytical model are required. Figure 1A shows that, in contrast to the measured viruses, EVs < 100 nm have a scattering cross section < 10 nm^2^ and are close to the trigger threshold and detection limit of the flow cytometer. Brittain et al. neither validated their procedure with reference particles having a scattering cross section < 10 nm^2^ nor confirmed their results with an analytical model. Except for particles with a scattering cross section < 10 nm^2^, our analytical model could describe the results of Brittain et al. well. Key assumptions of our model were already discussed in detail^10^. In short, our model assumes that the optical configuration of the flow cytometer is well-known, which was confirmed by the accurate (R^2^ = 0.9999) description of the beads data. Regarding the particles, our model assumes spherical particles, which was confirmed for EVs, because EVs < 500 nm in plasma are spherical^24^. In addition, our model assumes knowledge of the RI distribution within EVs. We introduced uncertainty ranges of the Ri distribution that are substantiated by literature values, because the RI distribution depends on the biochemical composition of EVs, which is still under investigation. We experimentally confirmed that our model well-describes scattering from hollow organosilica beads, which have an RI distribution resembling EVs^25^. However, similar to our model, the RI of the shell of hollow organosilica beads is isotropic, whereas the phospholipid membrane of EVs has different RIs in the radial and transverse directions, causing anisotropic scattering^26^. Although models are available to take into account anisotropic scattering of core–shell particles, further investigation is required to confirm whether anisotropic scattering of EVs is experimentally observable by flow cytometry^27^.

In sum, by two measurement artefacts and a statistical artefact, Brittain et al. overestimated the light scattering signals of EVs while underestimating the diameter. Consequently, the solid particle equivalent RI of 1.61 of 65 nm EVs is an overestimation. In turn, the claimed sensitivity of the CytoFLEX in terms of the diameter of plasma-derived EVs of 12 nm (p. 7), 30 nm (p. 8), 33 nm (p. 7), 65 nm (pp. 1, 8) are underestimations. Based on our own model and data, the smallest detectable EV diameter of the CytoFLEX ranges from 84 to 113 nm, depending on the exact RI distribution of EVs (Fig. 1A). In addition, we found a solid particle equivalent RI of 65 nm EVs between 1.402 and 1.475. Measurements of the true RI distribution of EVs smaller than 100 nm require a sensitive, calibrated instrument, a validated procedure, and confirmation of results with an analytical model.

## References

[1] Brittain GC (2019). A novel semiconductor-based flow cytometer with enhanced light-scatter sensitivity for the analysis of biological nanoparticles. Sci. Rep..

[2] Chen, Y. Q. *Flow Cytometer. US 10,209,174 B2*, 1–69 (2019).

[3] Li T (2016). Immuno-targeting the multifunctional CD38 using nanobody. Sci. Rep..

[4] Colombo M (2016). Tumour homing and therapeutic effect of colloidal nanoparticles depend on the number of attached antibodies. Nat. Commun..

[5] Hercher M, Mueller W, Shapiro HM (1979). Detection and discrimination of individual viruses by flow cytometry. J. Histochem. Cytochem..

[6] Steen HB (2004). Flow cytometer for measurement of the light scattering of viral and other submicroscopic particles. Cytometry A.

[7] Zhu S (2014). Light-scattering detection below the level of single fluorescent molecules for high-resolution characterization of functional nanoparticles. ACS Nano.

[8] van der Pol E, van Gemert MJC, Sturk A, Nieuwland R, van Leeuwen TG (2012). Single vs. swarm detection of microparticles and exosomes by flow cytometry. J. Thromb. Haemost..

[9] van der Pol E (2018). Standardization of extracellular vesicle measurements by flow cytometry through vesicle diameter approximation. J. Thromb. Haemost..

[10] de Rond L, Coumans FAW, Nieuwland R, van Leeuwen TG, van der Pol E (2018). Deriving extracellular vesicle size from scatter intensities measured by flow cytometry. Curr. Protoc. Cytom..

[11] van der Pol E (2018). Absolute sizing and label-free identification of extracellular vesicles by flow cytometry. Nanomed. Nanotechnol. Biol. Med..

[12] van der Pol E (2014). Particle size distribution of exosomes and microvesicles determined by transmission electron microscopy, flow cytometry, nanoparticle tracking analysis, and resistive pulse sensing. J. Thromb. Haemost..

[13] Van Manen H-JJ (2008). Refractive index sensing of green fluorescent proteins in living cells using fluorescence lifetime imaging microscopy. Biophys. J..

[14] Konokhova AI (2012). Light-scattering flow cytometry for identification and characterization of blood microparticles. J. Biomed. Opt..

[15] Konokhova AI (2016). Super-resolved calibration-free flow cytometric characterization of platelets and cell-derived microparticles in platelet-rich plasma. Cytometry A.

[16] Ducharme D, Max JJ, Salesse C, Leblanc RM (1990). Ellipsometric study of the physical states of phosphatidylcholines at the air-water interface. J. Phys. Chem..

[17] Barer R, Tkaczyk S (1954). Refractive index of concentrated protein solutions. Nature.

[18] Maltsev VP, Hoekstra AG, Yurkin MA (2011). Optics of white blood cells: Optical models, simulations, and experiments. Exp. Technol..

[19] Chen C (2017). Multiparameter quantification of liposomal nanomedicines at the single-particle level by high-sensitivity flow cytometry. ACS Appl. Mater. Interfaces.

[20] Hand DB (1935). The refractivity of protein solutions. J. Biol. Chem..

[21] Anderson W, Kozak D, Coleman VA, Jämting ÅK, Trau M (2013). A comparative study of submicron particle sizing platforms: Accuracy, precision and resolution analysis of polydisperse particle size distributions. J. Colloid Interface Sci..

[22] van der Pol E (2010). Optical and non-optical methods for detection and characterization of microparticles and exosomes. J. Thromb. Haemost..

[23] Dragovic RA (2011). Sizing and phenotyping of cellulars vesicles using nanoparticle tracking analysis. Nanomed. Nanotechnol. Biol. Med..

[24] Arraud N (2014). Extracellular vesicles from blood plasma: Determination of their morphology, size, phenotype and concentration. J. Thromb. Haemost..

[25] Varga Z (2018). Hollow organosilica beads as reference particles for optical detection of extracellular vesicles. J. Thromb. Haemost..

[26] Lange B, Aragón SR (1990). Mie scattering from thin anisotropic spherical shells. J. Chem. Phys..

[27] Bohren CF (1975). Scattering of electromagnetic waves by an optically active spherical shell. J. Chem. Phys..

